# MIP-4 is Induced by Bleomycin and Stimulates Cell Migration Partially via Nir-1 Receptor

**DOI:** 10.1155/2024/5527895

**Published:** 2024-08-02

**Authors:** M. Pacurari, I. Cox, A. N. Bible, S. Davern

**Affiliations:** ^1^ Department of Biology College of Science Engineering and Technology Jackson State University, Jackson, MS 39217, USA; ^2^ RCMI Center for Environmental Health College of Science Engineering and Technology Jackson State University, 1400 Lynch Street, 18750, Jackson, MS 39217, USA; ^3^ Environmental Science PhD Program College of Science Engineering and Technology Jackson State University, Jackson, MS 39217, USA; ^4^ Biosciences Division Oak Ridge National Laboratory, Oak Ridge, TN 37831, USA; ^5^ Radioisotope Science and Technology Division Oak Ridge National Laboratory, Oak Ridge, TN 37831, USA

## Abstract

**Background:**

CC-chemokine ligand 18 also known as MIP-4 is a chemokine with roles in inflammation and immune responses. It has been shown that MIP-4 is involved in the development of several diseases including lung fibrosis and cancer. How exactly MIP-4 is regulated and exerts its role in lung fibrosis remains unclear. Therefore, in the present study, we examined how MIP-4 is regulated and whether it acts via its potential receptor Nir-1.

**Materials and Methods:**

A549 cells were grown and maintained in DMEM : F12 (1 : 1) and supplemented with 10% FBS and 1000 U of penicillin/streptomycin and maintained as recommended by the manufacturer (ATCC). Cell migration and invasion, immunohistochemistry (IHC), Western blot, qPCR, and siRNA Nir-1 were used to determine MIP-4 regulation and its role in cell migration.

**Results:**

Cell migration was increased following stimulation of cells with recombinant (r) MIP-4 and bleomycin (BLM), whereas quenching rMIP-4 with its antibody (Ab) or addition of the Ab to BLM or H_2_O_2_ diminished rMIP-4-induced cell migration. Along with cell migration, rMIP-4, BLM, and H_2_O_2_ induced the formation of actin filaments dynamic structures whereas costimulation with MIP-4 Ab limited BLM- and H_2_O_2_-induced effects. MIP-4 mRNA and protein were increased by BLM and H_2_O_2_, and the addition of its Ab significantly reduced treatments effect. Experiments with siRNA investigating whether Nir-1 is a potential MIR-4 receptor indicated that the inhibition of Nir-1 decreased cell migration/invasion but did not totally inhibit rMIP-4-induced cell migration.

**Conclusion:**

Therefore, our data indicate that MIP-4 is regulated by BLM and H_2_O_2_ and costimulation with its Ab limits the effects on MIP-4 and that the Nir-1 receptor partially mediates MIP-4's effects on increased cell migration. These data also evidenced that MIP-4 is regulated by fibrotic and oxidative stimuli and that quenching MIP-4 with its Ab or therapeutically targeting the Nir-1 receptor may partially limit MIP-4 effects under fibrotic or oxidative stimulation.

## 1. Introduction

CC-chemokine ligand 18 also known as MIP-4 is a member of the large CC chemokines family and has been found to play a role in many diseases, including disease of the lungs such as fibrosis and cancer. MIP-4 expression is high in the lungs, lymph nodes, and placenta as well as in monocytes, macrophages, and immature dendritic cells [1–4]. As a secreted protein, MIP-4 appears to modulate many biological processes including inflammation, immune responses, and cell migration [5, 6]. *In vitro* studies show that MIP-4 induces T cells, B cells, and dendritic cells (DCs) migration [7, 8]. Moreover, human lung and dermal fibroblasts exposed to MIP-4 express high levels of collagen [9]. How exactly MIP-4 is regulated and exerts its biological effects is not well understood yet. Several receptors have been investigated as potential receptors in conveying MIP-4 signaling. A study by Islam et al. found CCR8 to bind MIP-4 and to activate downstream G-protein-coupled signaling and activation of cell chemotaxis [10]. Another study by Chen et al. identified PIRPNM3 as a receptor for MIP-4 that mediated the invasion and metastasis of breast cancer xenografts [11]. Although MIP-4 is a chemokine produced by M2 macrophages, lung fibroblasts as well as other several types of cancer cells produce MIP-4 [9, 12, 13]. MIP-4 was initially discovered as a pulmonary CC chemokine, and Pardo et al. found that MIP-4 is one out of many chemokines to be invariably increased in hypersensitivity pneumonitis and idiopathic pulmonary fibrosis compared to control lungs [14]. MIP-4 was found to be mainly produced by interstitial inflammatory cells and alveolar epithelial cells [14]. Serum analysis from patients with chronic obstructive pulmonary disease (COPD) showed high levels of MIP-4 compared to healthy controls and was correlated with clinic-pathological characteristics of the disease [15, 16]. *In vitro* studies showed that lung and skin fibroblasts exposed to MIP-4 stimulated the production of collagen [17]. In the present study, we investigated the regulation of MIP-4 and the role of Nir-1 in MIP-4-induced cell migration of lung adenocarcinoma A549 cells.

## 2. Materials and Methods

### 2.1. Chemicals

Recombinant (r) MIP-4, and MIP-4 antibody (Ab), H_2_O_2_ 30% (w/w), bleomycin (BLM), and FSL-1 were purchased from Millipore Sigma Aldrich and stored as recommended by the manufacturer. The siRNA transfection medium and siRNA Nir-1 were purchased from Santa Cruz Biotechnology and prepared and stored as recommended by the manufacturer.

### 2.2. Cell Culture

Human lung alveolar epithelial A549 cells were obtained from the American Type Culture Collection (ATCC, Manassas, VA) [18]. Cell cultures were maintained according to manufacturer's recommendations (ATCC). In brief, the cells were grown in DMEM : F12 medium (1 : 1, ATTC) supplemented with 10% fetal bovine serum (FBS) and streptomycin/neomycin (10,000/100 units, ATCC) in an incubator at 37°C with 5% CO_2_.

### 2.3. siRNA Transfection

The cells were transiently transfected with Nir-1 siRNA at a final concentration of 100 nM using siRNA transfection medium (Santa Cruz Biotechnology) according to the manufacturer's recommendations for 24 h followed by treatments.

### 2.4. RNA Isolation

Total RNA was extracted using Trizol® according to the manufacturer's protocol (Invitrogen, CA) [19, 20]. To ensure a good RNA quality, the integrity and quality of the total RNA were evaluated using 28S/18S ratio and a visual image of the 28S and 18S bands was evaluated on the 2100 Bioanalyzer (Agilent Technologies, Santa Clara, CA). The concentration of the total RNA was assessed using the NanoDrop-1000 spectrophotometer (NanoDrop Technologies, Germany).

### 2.5. Quantitative Real-Time (q)PCR

qPCR for target genes was determined using total RNA, and cDNA was generated using a High-Capacity cDNA Reverse Transcription kit and TaqMan gene expression assays (Applied Biosystems Inc., CA). The levels of MIP-4 and 18S mRNA were measured using SYBR-Green Master mix (Sigma Millipore) and gene specific primers according to manufacturer's protocol (Sigma Millipore). All qRT-PCR reactions were performed on 7500 instruments (Applied Biosystems Inc.). In the qRT-PCR analysis of genes, the dissociation curve showed the absence of a secondary peak, indicating the absence of primer dimer. The expression level of each gene was determined by the following formulas: fold change = 2−ΔΔ*Ct*, where Δ*Ct* (cycle threshold) =  *Ct*_target gene_ − *Ct*_endogenous control gene_, and ΔΔ*Ct* = Δ*Ct*_treated sample_ − Δ*Ct*_control sample_. 18S was used as an endogenous control gene [19, 20].

### 2.6. Wound Scratch Assay

Wound-healing assay was performed according to the methods previously described [19, 20]. A549 cells were grown on cover slips to a confluent monolayer and then scratched to form a 100 *μ*m “wound” using 100 µL sterile pipette tips. The cells were treated with PBS, rMIP-4, MIP-4 antibody (Ab), rMIP-4 + Ab, H_2_O_2_, or H_2_O_2_ + Ab in serum-free media for 24 h, washed one time with PBS, and then fixed in 4% paraformaldehyde. Samples were imaged using an Olympus IX70 microscope (Olympus Optical Co., Ltd, Japan) equipped with a Retiga 2000R FAST camera (Qimaging, Canada). Images were acquired using SimplePCI software (Compix Inc., Sewickley, PA).

### 2.7. Immunofluorescence

Immunofluorescence was used to detect the presence of proteins [19, 20]. In brief, the cells were grown on cover slips and treated for 24 h. After the treatments, the cells were fixed in 4% paraformaldehyde and permeabilized in 0.1% TritonX-100/PBS. Cells were incubated with 5% bovine serum albumin (BSA)/PBS/0.1% TritonX-100 for 2 h at room temperature and then incubated with respective antibodies (Sigma Aldrich, Thermo Fisher Scientific, Carlsbad, CA) in 5% BSA/PBS/0.1% TritonX-100 at 1 : 500 dilution overnight at 4°C. After primary incubation, the cells were washed three times with PBS and then incubated with an Alexa-Fluor®-488-conjugated secondary antibody (Molecular Probes, Life Technologies, MA)/5% BSA/PBS/0.1% TrtionX-100 at a dilution of 1 : 1000 for 1 hour in the dark. After secondary antibody incubation, the cells were washed with PBS three times for 5 min each time and mounted with a mounting antifade solution containing DAPI (Molecular Probes, Eugene, OR). Images were taken with an Olympus epifluorescence microscope (Center Valley, PA).

### 2.8. Western Blot

After treatments, the cells were lysed in 1X SDS lysis buffer (50 mM Tris-HCl, pH 6.8, 2% SDS, and 10% glycerol). Total protein was quantified by the BCA method. *β*-mercaptoethanol was added to lysates to a final concentration of 100 mM. Equal amounts of total protein were separated by 4–12% SDS-PAGE and transferred to a PVDF membrane. Membranes were blocked with 5% nonfat milk in 1X PBS containing 0.05% Tween-20 for 1 h at room temperature. Membranes were then incubated with primary antibody at 4°C overnight. After the incubation, the membranes were washed three times in 1X PBS with 0.05% Tween-20 for 10 min and then incubated for 1 h at room temperature with horseradish peroxidase (HRP)-conjugated goat anti-mouse IgG in 5% nonfat milk/1X PBS/0.05% Tween-20. Membranes were then washed five times for 10 min in 1X PBS with 0.05% Tween-20 and the proteins were visualized using an ECL Chemiluminescence Kit (Millipore, MA). The relative protein level was determined after normalization to *ꞵ*-actin [20].

### 2.9. Transwell Cell Migration/Invasion Assay

The migration assay was performed in a modified 24-well (8 *µ*m) Boyden chamber and according to the manufacturer's protocol (BD Biosciences, Franklin Lakes, NJ) with a slight modification [20]. The top insert chamber of the Transwell was loaded with 0.3 ml of 1 × 10^6^ cells/ml in serum-free media without or with rMIP-4, siRNA-Nir-1, H_2_O_2_, or combinations as shown in the figure. The bottom chamber of the insert was loaded with 0.5 ml of 0.1% growth medium as a chemoattractant, followed by incubation at 37°C in 5% CO_2_ for 48h. Cell migration assay results were analyzed according to manufacturer's protocol. After 48 h, the upper surface of the top insert was swabbed with cotton-tipped applicators to remove nonmigratory cells. The migrated cells were stained with 0.1% crystal violet followed by dye elution in 10% acetic acid. A microplate reader was used to measure the optical density (OD560 nm) of the eluted solutions to determine the migration values [20]. The mean values were obtained from four individual experiments.

### 2.10. Data Analysis

The statistical significance of effects and differences was determined using a two-way ANOVA. Student's *t-*test was used to compare the differences between groups. Statistically significant was considered at *p* ≤ 0.05.

## 3. Results

### 3.1. Recombinant MIP-4 Increases Cell Migration towards Wound Healing

**Figure 1:**
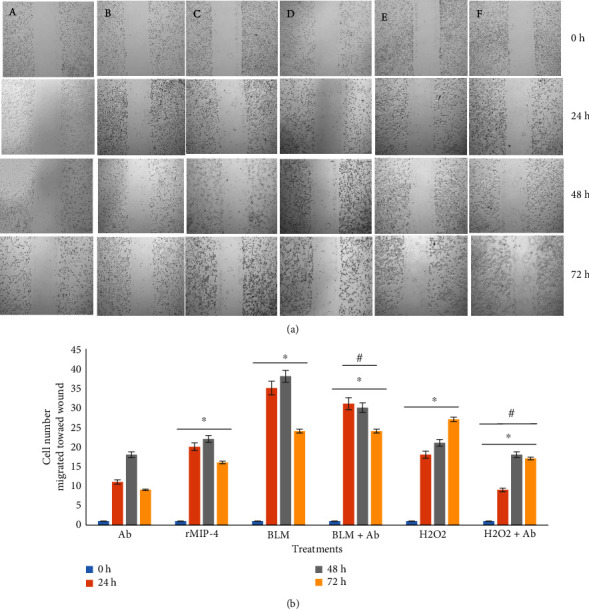
Cell migration in wound-healing assay. (a) The cell monolayer was wounded using scratch wound assay and exposed to (A) MIP-4 antibody (Ab), (B) rMIP-4, (C) BLM (30 *µ*g/ml), (D) BLM + Ab, (E) H_2_O_2_ (100 *µ*M), and (F) H_2_O_2_ + Ab. Cell migration towards wound healing was observed after 24, 48, or 72 h as described in materials and methods. One representative micrograph of scratch assay is shown. (b) Scratch assay quantification showing counted cells migrating towards wound healing. Data are presented as the mean ± SEM, *n* = 3. Statistically significant, ^*∗*^Ab (control) vs. treatment (trt) at respective time point, ^#^trt vs. trt + Ab, *t-*test, ANOVA, *p* ≤ 0.05.

To assess whether rMIP-4, BLM, H_2_O_2_, or pretreatment with MIP-4 Ab induce alveolar epithelial cells migration towards wound healing, the cells monolayers were subjected to a wound scratch and then exposed to rMIP-4, BLM, and H_2_O_2_ alone or in combination with Ab for 24, 48, or 72 h (Figure 1) and the number of cells migrating towards the wound were counted (Figure 1(b)). All treatments significantly stimulated cell migration towards wound vs. control (Ab) whereas rMIP-4 and BLM induced cell migration and the highest migration was observed after 48 h of exposure, whereas H_2_O_2_ induced cell migration over the entire tested period of 24−72 h (Figure 1(b)). Cotreatment of BLM with the Ab limited BLM-induced cell migration up to 48 h whereas for the 72 h time point, the Ab effect was not different vs. no Ab (Figure 1(b)). H_2_O_2_-induced cell migration was significantly limited in the presence of Ab for at all time points (Figure 1(b)).

### 3.2. Recombinant MIP-4 Stimulates Remodeling of Actin Filaments

**Figure 2:**
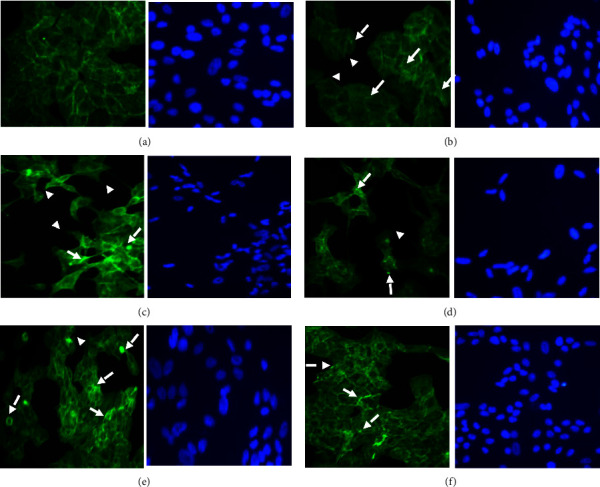
Actin filaments remodeling in A549 cells exposed to (a) MIP-4 Ab, (b) rMIP-4, (c) BLM (40 *µ*g/ml), (d) BLM + Ab, (e) H_2_O_2_ (100 *µ*M), and (f) H_2_O_2_ + Ab after 72 h as described in materials and methods. One representative micrograph is shown. Green = FITC-phalloidin staining of actin filaments; blue = DAPI staining of nuclei. Fluorescent microscopy was used to analyze the integrity of actin filament. Arrow = stress fiber (SF); broken arrow = focal adhesion (FA); arrow head = filopodium.

Cell migration is coordinated by changes in actin filaments (AFs). Here, we sought to determine whether stimulation with rMIP-4 induces reorganization of AF. AF reorganization was determined using FITC-phalloidin staining and fluorescent microscopy. It was found that in cells treated with the MIP-4 Ab, actin stress fibers (SFs) appeared in bundles at the cell periphery between cells in the monolayer (Figure 2(a),). In the presence of rMIP-4, AF appeared to undergo dynamic changes with SF forming across cell surface as well at the cell periphery between cells in the monolayer (Figure 2(b), arrow). Moreover, rMIP-4 induced the formation of focal adhesions (FAs) (Figure 2(b), broken arrow) and some cells exhibited filopodium (Figure 2(b), arrow head). BLM induced profound AF reorganization with the formation of SF, FA, and filopodium (Figure 2(c)); however, AF changes were attenuated in samples cotreated with MIP-4 antibody and BLM where FA adhesions appeared prominent (Figure 2(d)). H_2_O_2_ also induced profound AF structural changes with formation of profound FA and SF (Figure 2(e)). In samples cotreated with MIP-4 antibody, and H_2_O_2_ cells displayed remodeling of AF with the formation of SF at the cell periphery between the cells in the monolayer and the formation of smaller FA (Figure 2(f)).

### 3.3. MIP-4 Expression

**Figure 3:**
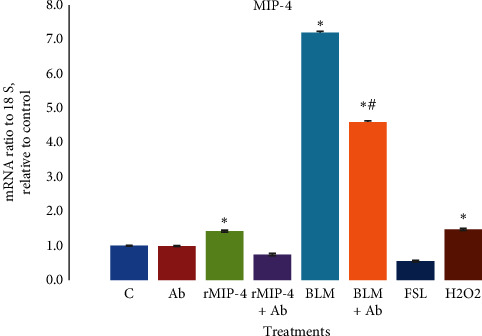
Mip-4 mRNA levels in the cells treated with: PBS or control (C), MIP-4 Ab (5 *µ*g/ml), rMIP-4 (1 *µ*g/mL), rMIP-4 + Ab (5 *µ*g/ml), BLM (40 *µ*g/ml), BLM + Ab, H_2_O_2_ (100 *µ*M), H_2_O_2_ + Ab for 24 h as described in Materials and Methods. The data are presented as mRNA fold change normalized to 18S relative to control sample. The data represent the mean ± SEM, *n* = 3. Statistically significant at *p* < 0.05, ANOVA, *t-test*, *n* = 3, ^*∗*^vs. control, ^#^vs. BLM.

**Figure 4:**
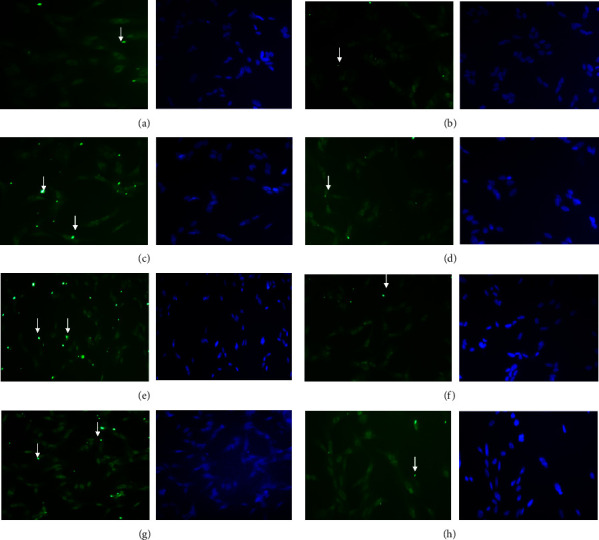
MIP-4 immunohistochemistry detection using MIP-4 immunofluorescence (green) and DAPI (nucleus). The cells were treated with (a) control, (b) MIP-4 Ab, (c) rMIP-4, (d) rMIP + Ab, (e) BLM, (f) BLM + Ab, (g) H_2_O_2_, and (h) H_2_O_2_ + Ab. The arrow points to immunofluorescence of MIP-4.

**Figure 5:**
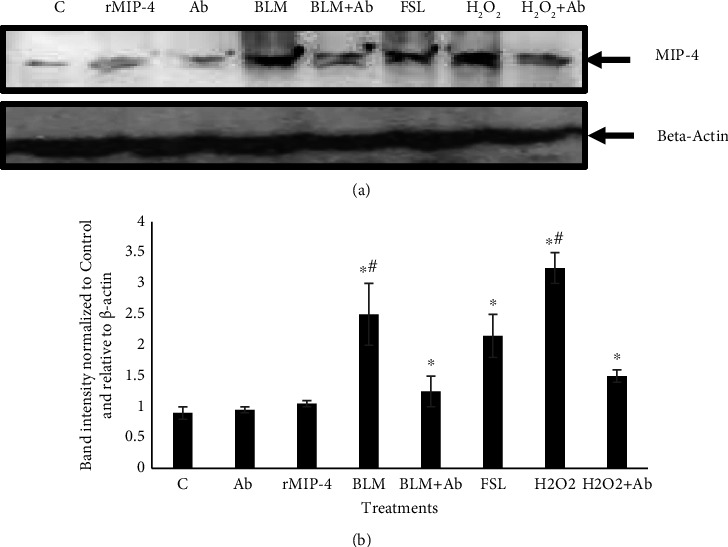
MIP-4 expression analysis. (a) MIP-4 protein level was determined by Western blot analysis. The protein amounts were quantified and normalized to *β*-actin in A549 cells treated as indicated in the figure. One representative blot is shown. (b) MIP-4 intensity analysis normalized to *β*-actin and relative to control untreated samples. The data represent the mean ± SEM, *n* *=* 3. Statistically significant: ^*∗*^vs. control, ^#^vs. treatment with Ab, ANOVA, *t-*test, *p* ≤ 0.05.

MIP-4 expression was analyzed using qPCR, immunohistochemistry, and Western blot. After performing qPCR analysis, we found no change in the MIP-4 mRNA levels in samples treated with MIP-4 Ab vs. control. However, MIP-4 mRNA was significantly increased by rMIP-4, BLM, and H_2_O_2_ treatments (Figure 3). In samples cotreated with BLM and MIP-4 Ab, there was a significant reduction in MIP-4 mRNA compared to BLM alone but still significantly higher compared to the control sample (Figure 3). MIP-4 mRNA was not regulated by FSL an agonist and activator of NF-*κ*B (Figure 3). Immunohistochemistry analysis showed that in control samples treated with PBS and MIP-4 Ab, MIP-4 immunodetection was weak with very few distinct foci of immunodetection (Figures 4(a) and 4(b)). MIP-4 immunodetection was increased in samples treated with rMIP-4, BLM, and H_2_O_2_ (Figures 4(c), 4(g), and 4(e)). In samples cotreated with BLM or MIP-4 Ab and H_2_O_2_, MIP-4 immunodetection was diminished (Figures 4(d), 4(f), and 4(h)). Western blot analysis showed strong bands immunodetection for MIP-4 in the presence of BLM, FSL, and H_2_O_2_ and the increase was significant compared to the control sample (Figure 5). However, MIP-4 band immunodetection was significantly reduced when the samples were treated with MIP-4 Ab and BLM or H_2_O_2_ (Figure 5). Western blot analysis indicates that BLM and H_2_O_2_ regulate the expression of MIP-4 and that quenching MIP-4 with its antibody abrogates its regulation. Moreover, we found that NF-*κ*B plays a role in regulating MIP-4 expression as we show that FLS, an agonist of NF-*κ*B, increased MIP-4 expression.

### 3.4. Cell Invasion/Migration

**Figure 6:**
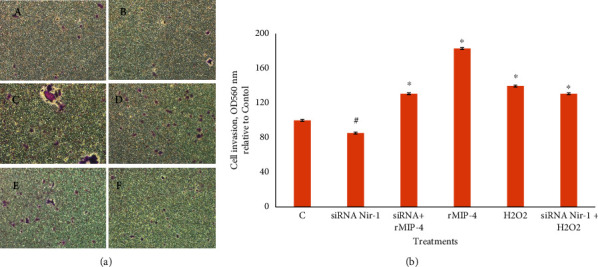
Analysis of cell invasion. (a) The cells were cultured in Boyden chamber inserts and treated as follows: (A) control, (B) siRNA Nir-1, (C) siRNA Nir-1 + rMIP-4, (D) rMIP-4, (E) H_2_O_2_ (100 *µ*M), and (F) siRNA Nir-  + H_2_O_2_ for 24 h and analyzed as described in materials and methods. Photomicrographs of invasive cells. (b) The cells were stained and quantified at OD560 nm after stained solution extraction according to the protocol. Data are OD560 nm relative to control cells. Statistically significant at *p* < 0.05, ANOVA, *t-*test, *n* = 3, ^*∗*^vs. control, ^#^vs. control and all treatments.

To determine whether rMIP-4 stimulates cell invasion and migration via the Nir-1 receptor, we employed inhibition of Nir-1 expression with siRNA for 24 h followed by exposing the cells to rMIP-4 or H_2_O_2_ for 24 h followed by cell fixation and removal of cells that did not invade the Transwell membrane on the other side. The cells that invaded the membrane on the other side were stained with crystal violet followed by imaging and absorbance measurement of released crystal violet upon elution of the stained membranes (Figure 6). Cell migration thorough the Transwell membrane was significantly reduced in the absence of Nir-1 but the addition or rMIP-4 to siRNA Nir-1 samples significantly increased cell invasion in the absence of rMIP-4 (Figures 6(a) panels (B) and (C) and 6(b)). The rMIP-4 and H_2_O_2_ significantly increased cell invasion. H_2_O_2_-induced cell invasion is mediated without Nir-1 (Figure 6).

## 4. Discussion

MIP-4, also known as CCL18, is a secreted protein involved in several biological processes, including immune response, inflammation, and cancer development [21–23]. Recent studies also indicated an MIP-4's role in pulmonary fibrosis [22]. Cai et al. [22] reported elevated levels of MIP-4 in the serum and bronchoalveolar lavage (BAL) fluid from patients with idiopathic pulmonary fibrosis compared to healthy control patients. In this study, we also found that MIP-4 is upregulated by pulmonary fibrosis experimental inducer BLM which places MIP-4 as a player in pulmonary fibrosis development. Nevertheless, therapeutic targeting of MIP-4 is impeded by the lack of an exact mechanism of signaling [24]. In the present study, we also investigated whether Nir-1 may work as a receptor for MIP-4, and we show that Nir-1 partially mediates rMIP-4 signaling on cell migration since siRNA Nir-1 inhibition only partially decreased rMIP-4-induced cell migration compared to rMIP-4 alone. Therefore, our study indicates complexity in finding the receptor that mediates MIP-4 biological effects.

Moreover, in the present study, we show that rMIP-4 enhances cell migration and these effects are abrogated in the presence of MIP-4 neutralizing Ab. We also show that BLM promotes cell migration towards a wound and that cotreatment with the MIP-4 neutralizing Ab inhibits cell migration induced by BLM. AF plays an important role in cell migration and we found that rMIP-4 induces actin filaments reorganization. More profound AF remodeling was present in cell treated with BLM with formation of profound SF and FA which were reduced in the presence of MIP-4-neutralizing Ab. Hydrogen peroxide induces actin filaments reorganization [25] and was used as a positive control for AF reorganization in the present study. The finding that rMIP-4 potentiates cell migration leads us to investigate whether MIP-4 is regulated by H_2_O_2_ and BLM. In the present study, we show that H_2_O_2_ and BLM increase the expression of MIP-4. BLM is a potent tumors inhibitor; however, it is also known to induce lung damage, including inflammation and fibrosis [26]. In the present study, we find that BLM increases MIP-4, which suggests that initially BLM is inflammatory, and if BLM treatment persists as is the case of its use in neoplasm treatment, the secondary effect of BLM is lung fibrosis, and as this study indicates, MIP-4 may represent one mechanism through which BLM leads to lung fibrosis. We also find that H_2_O_2_ is also a potent inducer of MIP-4. H_2_O_2_ is a well-known inducer of cell migration and inflammation, and MIP-4 appears to be involved in the signaling of H_2_O_2_. Moreover, we also show that NF-*κ*B plays a role in MIP-4 expression. We find that NF-*κ*B agonist, FSL, increased MIP-4 expression. NF-*κ*B is a well-known transcription factor involved in the regulation of numerous biological responses including cell migration [27]. Therefore, our finding that MIP-4 increases cell migration and its regulation by NF-*κ*B indicates complex MIP-4 regulation and its biological responses.

How MIP-4 exerts signaling on cells is less known. Previous studies indicate that CCL-18, also known as MIP-4, has many receptors among them Nir-1. Nir-1 is a homologue of mammalian lipid transfer proteins (LTPs) of phosphatidylinositol transfer proteins (PITPNM3/RdgB*α*III) which also includes Nir-2 (PITPNM1/RdgB*α*I) and Nir-3 (PITPNM2/RdgB*α*II), both of which facilitate phosphatidyl inositol (PI) signaling [28]. Some studies suggest that Nir-1 has similar functions to Nir-2 and Nir-3 by positively modulating PI cycling from the endoplasmic reticulum (ER) to the plasma membrane (PM), thus regulating PIP2 replenishment within the PM [29]. Nir-1's role in human disease is still unclear; however, Nir-1 has been linked to human disease. For example, Nir-1 contains mutations in patients with autosomal dominant cone dystrophy, whereas in various human cancer cell lines, Nir-1 promotes metastasis [30–32]. In the present study, we find that Nir-1 is a receptor for MIP-4; however, it is not the only receptor for MIP-4 signaling. We show that inhibition of Nir-1 partially decreases rMIP-4-induced cell migration. Moreover, we also find that MIP-4-induced cell migration is limited when rMIP-4 is quenched with its Ab.

In conclusion, we find that Nir-1 contributes to MIP-4 signaling; however, Nir-1 is not the sole receptor for MIP-4, suggesting that other receptors also mediate MIP4 signaling, at least when Nir-1 is not expressed.

## Data Availability

The data used to support the findings of this study are available from the corresponding author upon reasonable request.
